# A comparative study on the influence of single and combined ultrasounds assisted flake graphite flotation

**DOI:** 10.1016/j.ultsonch.2023.106551

**Published:** 2023-08-07

**Authors:** Shaoqi Zhou, Zheng Tong, Lisha Dong, Xiangning Bu, Chao Ni, Guangyuan Xie, Muidh Alheshibri

**Affiliations:** a Key Laboratory of Coal Processing and Efficient Utilization (Ministry of Education), School of Chemical Engineering and Technology, China University of Mining and Technology, Xuzhou 221116, China; b Western Australian School of Mines: Minerals, Energy and Chemical Engineering, Curtin University, Kalgoorlie, WA 6430, Australia; c Department of General Studies, Jubail Industrial College, P. O. Box 10099, Jubail Industrial City 31961, Saudi Arabia

**Keywords:** Graphite flotation, Dual-frequency ultrasound, Cavitation intensity, Parametric optimization, Ultrasonic horn, Ultrasonic bath

## Abstract

Ultrasound has emerged as a promising technique for improving the mineral flotation performance. However, limited research exists regarding the influence of different ultrasound types on the flotation process. Specifically, the impact of combined ultrasound and the comparison of horn- and bath-type ultrasounds on flotation have not been fully investigated. To address this knowledge gap, a comprehensive study to explore the effects of different ultrasonic pretreatments on the flotation of flake graphite was conducted. A Box-Behnken design is employed to analyze the effects of combined ultrasound on graphite flotation. By characterizing the properties of graphite samples before and after the ultrasonic treatment, the aim is to elucidate the mechanism underlying the impact of ultrasound on graphite flotation. The experimental results indicated that the ultrasonic cavitation intensity exerted a significant influence on the graphite flotation recovery. Both horn- and bath- type ultrasounds contributed to flotation, but horn-type ultrasound demonstrated a more pronounced effect, leading to a 7% increase in flotation recovery, whereas bath-type ultrasound resulted in only a 2% increase. Furthermore, the cavitation intensity of combined ultrasound was found to be higher than that of single-frequency ultrasound in the same duration. However, the performance of graphite flotation was better with short duration combined ultrasound pretreatment, while the opposite trend was observed for a long duration ultrasound pretreatment. These findings may inform the development of more efficient and effective ultrasonic pretreatments for flotation separation processes.

## Highlights

• Ultrasonic parameters of graphite flotation were optimized by Box–Behnken design.
• Horn-type ultrasound produced a better flotation efficiency than that of bath-type.
• Dual-frequency ultrasound was first employed for graphite flotation.
• Related mechanisms are cavitation intensity and formed bubble-particle aggregates.

## 1 Introduction

Mineral flotation is a vital separation technique [1], [2], and diverse methods have been explored to enhance its efficiency, including the utilization of magnetic field [3], sound field [4], [5], electric field [6], [7], etc. Ultrasound technology has gained significant attention due to its accelerating development and wide applications. In these applications, acoustic cavitation occurs when ultrasonic waves propagate in liquids, accompanied by strong shock waves and micro-jets creating strong mechanical agitation between interfaces [8], [9]. In addition, this mechanical agitation effect can break through the laminar boundary layer, providing an ideal environment for interfacial reactions in mineral flotation [10]. Consequently, recent years have witnessed a surge in the research devoted to exploring the utilization of ultrasound to enhance the mineral flotation performance.

Videla et al. [11] employed the ultrasonic pretreatment on copper sulfide tailings and reported an improved copper flotation recovery of 3.5%. Authors attributed this enhancement to acoustic cavitation-induced removal of slime coatings on particle surfaces, which facilitated reagent adsorption. Cilek and Ozgen [12] placed an ultrasound probe in the froth zone and investigated the flotation performance of a complex sulphide ore. They observed a significant improvement in the flotation separation selectivity and efficiency owing to the ultrasonic treatment. Huang et al. [13] explored the impact of ultrasound on the properties and microstructure of benzohydroxamic acid (BHA) solution, as well as its effect on the flotation separation of scheelite and calcite. The study revealed that ultrasonic treatment of BHA led to reduced surface tension and adsorption efficiency, which significantly enhanced the flotation rate of scheelite. However, the effect on the flotation rate of calcite was found to be less pronounced.

In addition to its applications in metallic ore flotation, ultrasound has gained significant attention for its wide use in the flotation of non-metallic ores and solid waste. For example, Altun et al. [14] employed the ultrasound to enhance the flotation cleaning of oil shales, reporting a decrease in ash content from 34.76% to 11.82% and with a combustible recovery of 82.66%. Authors attributed this improvement to the ultrasonic pretreatment to help liberate organic and inorganic entities as well as clean impurities from the oil shale pores. Mao et al. [15] investigated the effects of conventional flotation and simultaneous ultrasonic flotation in the pulp/froth zones on the separation selectivity and kinetics of high-ash lignite flotation. Results showed that simultaneous ultrasonic treatment in the pulp zone significantly improves lignite flotation selectivity, while the use of ultrasonic treatment in the froth zone does not improve lignite selectivity. They explained this result by highlighting that ultrasonic treatment in the pulp zone can eliminate coal particle slime coatings, while the foam layer generated after ultrasonic treatment in the froth zone intensifies water recovery and gangue minerals entrainment. Barma et al. [16] found that low-frequency ultrasonic pretreatment of graphite slurry can significantly improve the recovery of flaky graphite from low-grade graphite ore. Authors ascribed this effect to the ultrasound pretreatment to help promote the liberation of graphite from gangue minerals.

Table 1 presents a comprehensive summary of the utilization of ultrasound in mineral flotation. The table indicates that ultrasound has been extensively applied in the flotation of various minerals, including non-ferrous metals, coal, graphite, gasification slag, oil shale, and others. Remarkably, the literature reports substantial variations in the ultrasound type, frequency, and application methods across different studies. Ultrasound types are classified into two main categories, namely the bath-type and horn-type, with generally low frequencies of <100 kHz. Only several scholars have employed high-frequency ultrasound in the mineral flotation. Application methods of ultrasound in flotation are broadly classified into two types: pre-conditioning treatment, where the pulp is pre-treated with ultrasound before flotation, and simultaneous treatment, where ultrasound is applied during the flotation process. Notably, the cavitation effect induced by the ultrasound is critical to its role in the mineral flotation. The rupture of cavitation bubbles generates high-speed shockwaves and micro jets, which can have powerful mechanical and chemical effects such as particle surface cleaning, particle crushing, reagent emulsification, and under specific conditions, micro-nanobubble generation. Therefore, the ultrasonic cavitation phenomenon plays a crucial role in the mineral flotation.

**Table 1:** Application of ultrasound in mineral flotation.

**Samples**	**Ultrasound Type**	**Ultrasound frequency (kHz)**	**Processing method**	**Reference**
Copper	Bath-type	20	Per-conditioning treatment and simultaneous treatment	Videla, et al. [11]
Copper–iron sulphide ore	Horn-type	20	Simultaneous treatment	Cilek and Ozgen [12]
Oil shales	Horn-type	20	Per-conditioning treatment	Altun, et al. [14]
Oxidized pyrite	Bath-type	28	Simultaneous treatment	Cao, et al. [17]
lead–zinc-copper ore	Bath-type	40	Per-conditioning treatment	Kursun [18]
Colemanite	Bath-type	35	Per-conditioning treatment	Ozkan and Gungoren [19]
Barite and chalcopyrite	Horn-type	20	Simultaneous treatment	Cilek and Ozgen [20]
High sulfur coal	Bath-type	59	Per-conditioning treatment	Zhang, et al. [21]
Coal	Horn-type	20	Per-conditioning treatment and simultaneous treatment	Mao, et al. [15]
Coal	Bath type	50 200 600	Simultaneous treatment	Chen, et al. [22]
Graphite	Bath-type	40	Per-conditioning treatment	Barma, et al. [16]
Gasification coal fine slag	Horn-type	20	Per-conditioning treatment	Wang, et al. [23]

Ultrasonic cavitation, a crucial physicochemical phenomenon, is subject to various influencing factors, such as ultrasound type, ultrasound time, ultrasonic intensity, and others, which result in different cavitation field characteristics. In the realm of ultrasonic flotation, researchers typically manipulate ultrasonic intensity and time to optimize the cavitation intensity and, therefore, the flotation efficiency. However, the application of single-frequency ultrasound has significantly constrained the potential of ultrasonic separation processes. Recently, dual-frequency ultrasound models have gained attention in fields such as chemical engineering and environmental engineering. For instance, researchers have reported a strong synergistic effect of dual-field ultrasound in degrading nitrobenzene [24] and methylene blue [25] in water. Ye, et al. [26] have developed a kinetic model for dual-frequency ultrasonic cavitation and investigated the dynamic evolution of bubbles under single- and dual-frequency ultrasound via numerical simulations, revealing that dual-frequency ultrasound enhances the cavitation effect significantly. Liu and Hsieh [27] have shown that under specific test conditions, dual-frequency ultrasound can generate bubbles at levels up to five times higher than those observed under single-frequency stimulation.

Currently, single-frequency ultrasound predominates in mineral flotation applications, while different researchers employ distinct types of ultrasound, including horn-type and bath-type ultrasound. Despite their shared purpose in the mineral flotation process, these two ultrasound variants exhibit divergent characteristics. The bath-type ultrasound exhibits a notably uniform sound field, characterized by an absence of dead angles that would impede sound wave propagation. In contrast, the horn-type ultrasound primarily concentrates its sound field in close proximity to the probe's location [28]. Combining the two types of ultrasound can complement each other. Moreover, studies in various fields have demonstrated the superior performance of combined ultrasound in the process intensification, because combined ultrasound can also increase the cavitation intensity.

However, to date, no studies have undertaken a comparative examination of the mechanisms underlying different types of ultrasound on flotation. Furthermore, no research has explored the potential impact stemming from the synergistic application of both ultrasound types on the flotation process, particularly in flake graphite flotation. Therefore, the present study aims to comprehensively investigate the influence of ultrasonic parameters, such as time, power, and combination mode, on the process of further ash content removal (i.e. further purification) applied to graphite concentrate. This investigation will encompass both single-frequency and combination ultrasound conditions to gain insights into their respective mechanisms on the flotation process.

## 2 Experimental and methodology

### 2.1 Materials and reagents

The flake graphite concentrate sourced from Heilongjiang province, China, is employed for all flotation experiments. The ash content of this graphite concentrate is about 7.50%, and the volatile content is about 1.02%, the carbon content in the raw ore is about 91.50%. Kerosene and secondary octanol from the Sinopharm Group Co. Ltd., China are used collector and frother, respectively. All tests were conducted in tap water at natural pH.

### 2.2 Flotation experiments

**Fig. 1:**
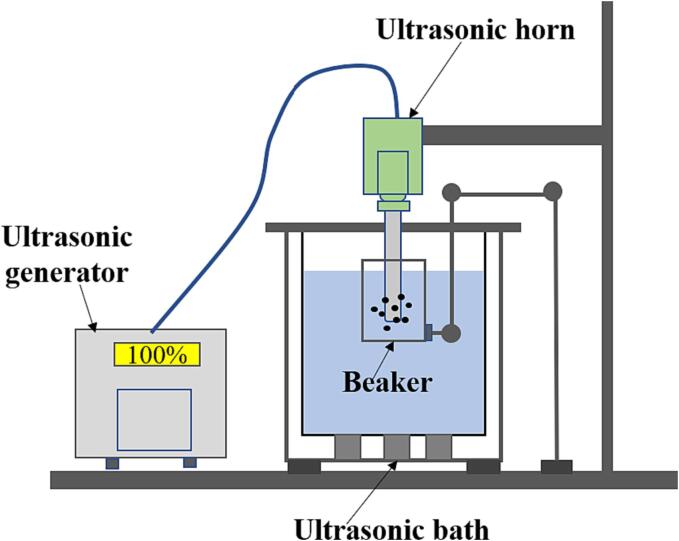
Schematic diagram of combined ultrasound.

This study employs a pre-conditioning treatment in which ultrasound is utilized during the slurry conditioning process prior to flotation. Specifically, the pre-conditioning method comprises two variants: single-frequency and combined ultrasonic pretreatment. The impact of ultrasonic time (ranging from 0 to 7 min) and ultrasonic intensity (varying between 0% and 100%) generated by two distinct ultrasound generators on the separation of flake graphite concentrate was assessed individually. The equipment used for the single-frequency ultrasound experiments consisted of a bath-type (40 kHz, 600 W, 100ST, Fuyang Technology Group Co., Ltd) and a horn-type (20 kHz, 800 W, VCX800, Sonics & Materials Inc). In the case of combined ultrasound, these two single-frequency ultrasound generators were merged and the impact of the two ultrasound combinations on graphite flotation was investigated using the response surface method. During the combined ultrasound treatment, the beaker containing the graphite slurry should be positioned within the bath-type ultrasonic tank, ensuring that the slurry level remains below the water level in the ultrasonic tank. Subsequently, immerse the ultrasonic probe three centimeters below the slurry level. Set the required parameter values for both ultrasound devices as per the experiment's specifications, and activate both devices simultaneously. After subjecting the pulp to the sound field for the designated duration, the devices were turned off. A schematic diagram of the experimental setup is presented in Fig. 1.

The flotation experiments were conducted in a 0.5 L flotation cell. Prior to each test, a quantity of 20 g of flake graphite was accurately weighed and added to a plastic beaker containing 200 mL of water. The mixture was then stirred with a glass rod at a constant rate (200 r/min) for one minute to optimize the dispersion of graphite in the water. The resulting graphite slurry was subjected to ultrasonic pre-treatment and subsequently transferred to the flotation tank for flotation. Following the flotation, the collected flotation concentrates and tailings were subjected to filtration, drying, ash-burning, and weighing, after which the carbon recovery was computed using the formula below:

(1) $$w_{c}=100-A_{d}-V_{d}$$

(2) $$\varepsilon =\frac{w_{c}\gamma_{i}}{\alpha }\times 100$$

where *w_c_*, *A_d_* and *V*_d_ represent the concentrate carbon content, ash content and volatile matter. *ε, γ*_i_, and *α* represent carbon recovery, concentrate recovery, and carbon content in the raw ore.

### 2.3 Box-Behnken design

The Box-Behnken design (BBD) was utilized to conduct the combined ultrasound tests, with three variables, namely bath-type intensity, horn-type intensity, and duraton of ultrasound treatment, investigated at three levels to determine their impact on the performance of flake graphite flotation. As has been reported in prior studies [29], [30], the levels were represented as + 1 (high), 0 (middle), and −1 (low). In the BBD experiment, it is essential to incorporate both the minimum and highest levels for each factor during the design phase, leading to the selection of power ratios at 20% and 100% to ensure a comprehensive analysis. The determination of the minimum processing time at 1 min and the maximum duration at 7 min is based on the findings obtained from the exploration of the single-frequency experiment. The specific levels of the three variables are presented in Table 2, while the values of the three factors for the 17 runs are listed in Table 3. The response surface data analysis and model building were conducted using Design-Expert V.8.0.6.1 software, with five replicates performed at the central point to evaluate the pure error, also known as random error. These errors are inevitable and cannot be eliminated by any predictive equation that assigns a predicted value for the dependent variable based on the value of the independent variable [30]. The carbon recovery of graphite was selected as the response variable.

**Table 2:** Variables and levels used in BBD.

Variables	Symbol	Level		
		−1	0	1
Horn-type intensity / %	A	20	60	100
Bath-type intensity / %	B	20	60	100
Ultrasound duration / min	C	1	4	7

**Table 3:** BBD experimental results.

Run number	A (Horn-type power / %)	B (Horn-type power / %)	C (Ultrasound duration / min)	Carbon recovery (%)
1	20	20	4	91.11
2	20	60	1	90.74
3	20	60	7	91.97
4	20	100	4	91.08
				
5	60	20	1	92.46
6	60	20	7	94.67
				
7	60	60	4	94.47
8	60	60	4	93.16
9	60	60	4	94.78
10	60	60	4	94.02
11	60	60	4	93.94
				
12	60	100	1	93.97
13	60	100	7	95.97
				
14	100	20	4	96.04
15	100	60	7	95.63
16	100	60	1	97.03
17	100	100	4	95.93

### 2.4 Characterizations of graphite

In this study, the variation of graphite particle size before and after treatment with ultrasound was assessed using focused beam reflection measurement (FBRM, G400, Mettler-Toledo Ltd., USA). The detailed operational procedure can be found in our prior studies [1], [31]. Furthermore, X-Ray diffraction analysis (D8 Advance, Bruker Company, Germany) was utilized to monitor any changes in the physical phase of graphite resulting from ultrasonic pretreatment. The element distribution and morphology of graphite were analyzed using a scanning electron microscope (SEM, SU8010, Hitachi High-Tech, Japan), with the test methods having been previously described [32].

### 2.5 Measurement of cavitation intensity

The iodine release method is a typical approach for measuring cavitation behavior in the ultrasonic field and has been widely used [33], [34], [35], [36]. Transient cavitation could be produced in potassium iodide solution under ultrasound treatment, and the heat (5000 K) generated by the collapse of transient cavitation bubbles can cause water vapor degradation within the cavitation bubble. The degree of cavitation bubble collapse serves as an intuitive representation of cavitation intensity. Moreover, the free radicals generated when the cavitation bubbles collapse will undergo a series of reactions with the potassium iodide solution, enabling the determination of cavitation intensity through the quantification of I_3_^−^ formed in the potassium iodide solution [33]. The chemical reaction involved are presented in equations 3–6. In this work, the concentration of I_3_^−^ was measured by UV spectrophotometry (EVOLUTION 350, Thermo Fisher Scientific, USA). Before tests, the linear relationship between different I_3_^−^ solutions and absorbance was first established. The detailed steps are as follows: Five iodine solutions were first prepared with concentrations ranging from 1 × 10^−7^ to 1 × 10^−3^ M. The characteristic peaks of I_3_^−^ were then determined by full spectral scanning. Finally, the absorbance of five different concentrations of I_3_^−^ solutions at fixed characteristic peaks (351.36 nm) was obtained to establish the linear relationship between concentration and absorbance, and the formula is y=1392.70x+0.0045.

(3) $${\text{H}}_{2}\text{O}\to \cdot \text{H}+\cdot \text{OH}$$

(4) $$2\text{OH}\cdot \to {\text{H}}_{2}{\text{O}}_{2}$$

(5) $${\text{H}}_{2}{\text{O}}_{2}+2\text{KI}\to 2\text{KOH}+{\text{I}}_{2}$$

(6) $${\text{I}}_{2}+{\text{I}}^{-}\to {\text{I}}_{3}^{-}$$

The procedure for assessing cavitation strength is as follows: A potassium iodide solution of 0.2 mol/L was prepared, from which 50 mL was withdrawn for each test and exposed to varying levels of sonication. After sonication, the sample absorbance was measured by employing a UV spectrophotometer under the designated characteristic peaks. Subsequently, the concentration of I_3_^−^ in the sample was calculated by utilizing a pre-established linear correlation between the concentration of I_3_^−^ and the absorbance. Greater cavitation intensity corresponded to higher I_3_^−^ concentration following ultrasonic radiation.

## 3 Results and discussions

### 3.1 Single-frequency ultrasound

**Fig. 2:**
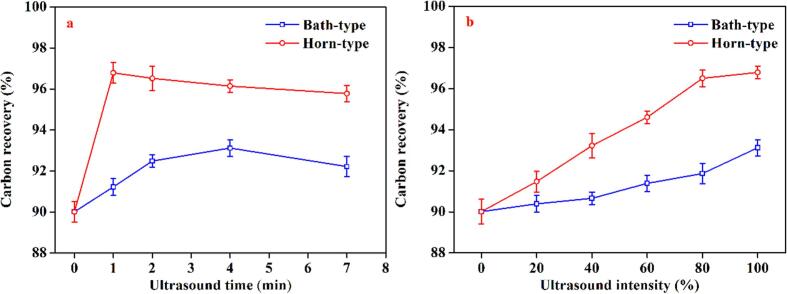
Effect of ultrasound time on the carbon recovery.

The present study investigated the impact of sonication duration on the carbon recovery of flake graphite using two types of ultrasonic generators (Fig. 2a). Irrespective of the type of ultrasonic generator employed, the carbon recovery of flake graphite demonstrated an increasing trend with sonication time followed by a subsequent decline, when the ultrasound intensity was fixed at 100%. Moreover, the flotation carbon recovery of horn-type pretreated graphite ore was observed to be consistently higher than that of bath-type. Specifically, a significant enhancement in the flotation carbon recovery of horn-type pretreated graphite was observed with an increase in ultrasonic pretreatment duration from 0 to 7 min. The maximum recovery of 96.79% was achieved with a pretreatment duration of 1 min, beyond which the carbon recovery showed a slight decline trend. These results suggest that prolonging the sonication treatment duration beyond the optimum point may not yield further improvement in carbon recovery, and instead, may even have an adverse effect on it. The possible reason for this phenomenon is that prolonged ultrasound exposure could severely affect the particle size distribution (PSD) of graphite, which may not favor the flotation process [37]. The impact of sonication on the PSD of graphite will be discussed in detail in the subsequent sections.

In comparison to the horn-type, the carbon recovery improvement observed for the bath-type in this graphite sample was relatively weak. The carbon recovery exhibited a gradual increase with increasing pretreatment time until it attained a peak value of 93.22% after the sonication for 4 min. Subsequently, the carbon recovery demonstrated a slight decline with further extension of the sonication time. This phenomenon can be attributed to the fact that a certain duration of sonication possesses a particle-cleaning effect [38], [39]. However, prolonged sonication can result in excessive dispersion of graphite particles, thereby compromising the carbon recovery.

The impact of different ultrasonic intensities on the flotation carbon recovery under optimal pretreatment conditions for both types of ultrasounds was analyzed, and results are illustrated in Fig. 2b. Analogous to the effect of pretreatment time, the carbon recovery demonstrated a gradual increase with the augmentation of ultrasonic intensity, regardless of the type of ultrasound, when the ultrasonic pretreatment time was fixed. Moreover, the carbon recovery of horn-type was consistently higher than that of bath-type. Specifically, for the horn-type, the carbon recovery showed a gradual increase from 90.01% to 96.79% as the ultrasonic intensity increased from 0% to 100%. For the bath-type, the carbon recovery increased from 90.01% to 93.22%. However, the enhancement in carbon recovery was relatively weak at lower ultrasonic intensities.

### 3.2 Combined-frequency ultrasound

The single-frequency experimental exploration has revealed that the attainment of an appropriate cavitation intensity is critical for improving graphite flotation. Combination of two types of ultrasounds has been extensively recognized as a promising approach for enhancing cavitation intensity. Accordingly, the effectiveness of the combined ultrasound approach on graphite flotation carbon recovery were analyzed through Box-Behnken Design (BBD) experiments. The outcomes of the 17 BBD trial runs are presented in Table 3. Four models, namely, linear, 2FI (interactive), quadratic, and cubic, were employed to fit the experimental results. The fitting results of the different models are summarized in Table 4. It is noteworthy that the linear model exhibited favorable adjusted R^2^ and predicted R^2^ values and is also recommended by Design-Expert. Table 5 was generated through significance optimization and analysis of variance (ANOVA) analysis.

**Table 4:** Summary of model statistics for the BBD experiments.

Source	Std. Dev.	R^2^	Adjusted R^2^	Predicted R^2^	PRESS
Linear	0.78	0.8666	0.8358	0.7468	15.07
2FI	0.79	0.8959	0.8334	0.5730	25.42
Quadratic	0.85	0.9160	0.8080	0.0225	58.19
Cubic	0.61	0.9746	0.8984		+

Note: PRESS (prediction error of square sum); + case (s) with leverage of 1.000.

**Table 5:** ANOVA analysis for the response surface.

Source	Sum of Squares	DF	Mean Square	F-Value	P-value Prob > F	Remarks
Model	51.59	4	12.90	19.50	< 0.0001	significant
A	48.66	1	48.66	73.56	< 0.0001	
B	0.89	1	0.89	1.35	0.2684	
C	2.04	1	2.04	3.08	0.1045	
AB	1.60 × 10^−3^	1	1.60 × 10^−3^	2.42 × 10^−3^	0.9616	
Residual	7.94	12	0.66			
Lack of Fit	6.43	8	0.80	2.13	0.2432	not significant
Pure Error	1.51	4	0.38			
Cor Total	59.53	16				

Notes: Values of “Prob > F” <0.05 indicate model terms are significant. Values greater than 0.10 indicate the model terms are not significant.

According to Tables 4 and 5, although R^2^_Adj_ = 0.8358 in the designed experiment was low, the R^2^ of the fitted model was significant at the considered confidence level because the very low probability (P < 0.05) indicated that the model was in good agreement with the experimental results [33]. The Model F-value of 19.50 implies the model is significant, because the F-value is greater than 0.001. The p-value is < 0.05, showing < 0.0001, which mean that there are only 0.01% of the total variation that could not be explained by the model because of noise. In this model analysis, the power of the two types of ultrasound devices (i.e., factor A and factor B), the duration of ultrasound, and the interaction between the two types of ultrasound devices were taken into consideration. In this case, factor A (horn ultrasound intensity) is significant model term. The lack of fit is the variation of the data around the fitted model and tests the adequacy of a model fit [40]. The “Lack of Fit F-value” of 2.13 implies the Lack of Fit is no statistically significant as p-value (0.243) is greater than 0.05, and the model can be successfully used for the prediction, because the lack of fit is insignificant. Based on the model fit, a predictive equation for graphite carbon recovery that incorporates the effects of the factors is given:

(7) $$R_{\mathrm{carbon}}=89.02+0.06\times A+9.09\times 10^{-3}\times B+0.17\times C-1.25\times 10^{-5}\times A\times B$$

The optimal values obtained by the optimization were: horn-type ultrasound intensity 100%, bath-type ultrasound intensity 100%, and ultrasound time of 7 min, the predicted value of flotation carbon recovery is 97.224% with a desirability of 0.837. To validate the model, graphite flotation tests were conducted under the predicted optimal experimental conditions. Under these conditions, the carbon recovery of this graphite is 95.43%, which is lower than the expected value, this may be because the higher cavitation intensity under this condition leads the graphite particle size to too fine.

**Fig. 3:**
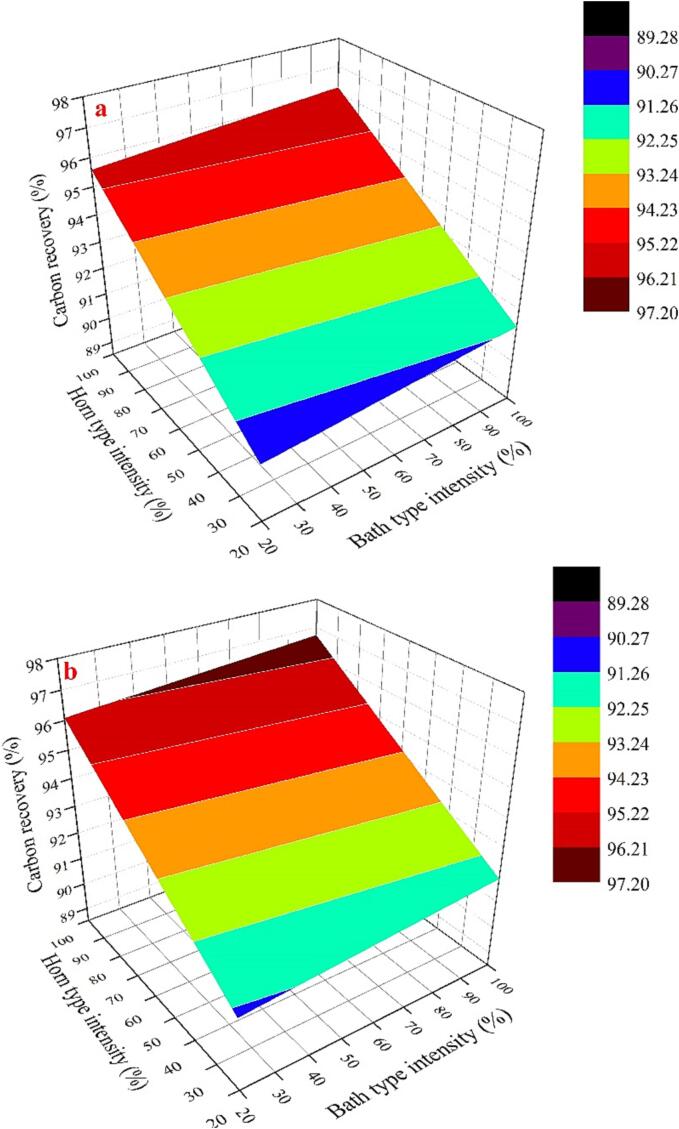

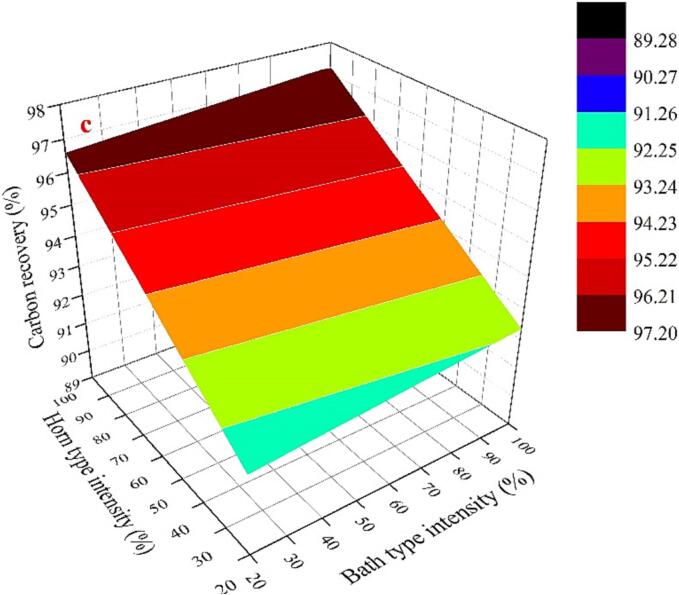
3D surface demonstrating the relationship between the intensity of combination of two ultrasounds and carbon recovery (a: 1 min, b:4 min, c:7 min).

Fig. 3 shows the effect of combination of two types of ultrasounds on the carbon recovery under different ultrasound treatment duration, respectively. Overall, the carbon recovery rate showed a linear relationship with the combined ultrasound intensity regardless of the duration of ultrasound treatment. It indicates that there is no cooperative effect of two types of ultrasounds on the carbon recovery rate. As can be seen from Fig. 3a, when the ultrasound time is 1 min, the slope of the response surface curves for both factor A (intensity of the horn-type ultrasound) and factor B (intensity of the bath-type ultrasound) showed an increasing trend. But the slope for the effect of factor A is much steeper than that of factor B. It indicates that factor A plays a dominant role in graphite carbon recovery compared to factor B, which is consistent with the results obtained from ANOVA analysis. As the sonication time increases, the response surface curves show the similar trend (Fig. 3a and b), except that the overall carbon recovery increases as the sonication time increases.

### 3.3 Mechanism

#### 3.3.1 Particle size

**Fig. 4:**
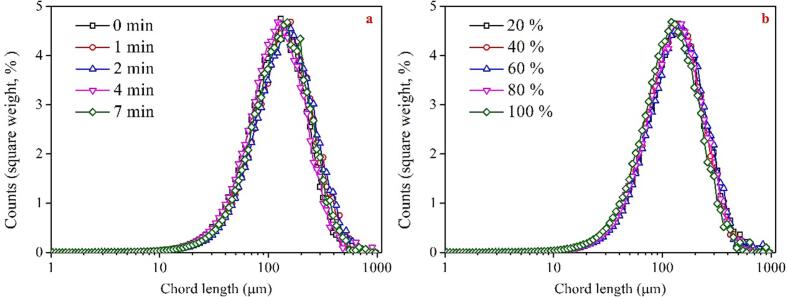
Effect of bath-type ultrasound parameters on the PSD of graphite (a: intensity of 100% with different treatment time; time of 4 min with different intensities).

The mechanical effects generated by the collapse of cavitation bubbles during ultra-sonication have been demonstrated to influence the properties of particles in the pulp. The properties of graphite particles are considered one of the crucial factors affecting flotation. To further elucidate the underlying mechanism of ultrasonic pretreatment methods affecting the graphite flotation, the PSD of graphite slurry after subjecting it to various types of ultrasonic pretreatment was investigated. Fig. 4a and b illustrates the effect of bath-type ultrasonic pretreatment on the PSD of graphite with different operating parameters. Notably, altering the sonication duration or intensity did not induce any significant changes in the PSD of graphite. Regardless of the presence or absence of ultrasonic pretreatment, the particle size of graphite exhibited a single-peak distribution with the peak appearing at around 150 μm, indicating that bath-type ultrasonic has a negligible effect on the graphite particle size.

**Fig. 5:**
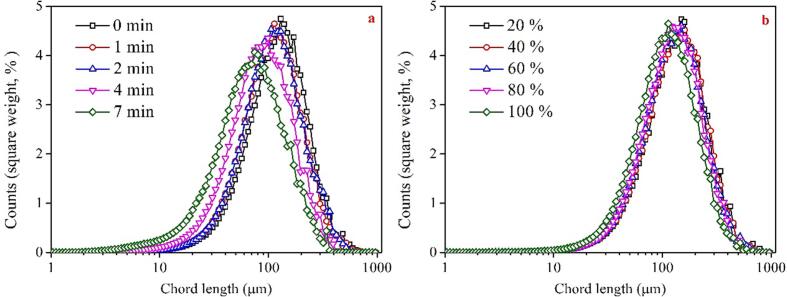
Effects of horn-type ultrasound parameters on the PSD of graphite (a: intensity of 100% with different treatment time; time of 1 min with different intensities).

In comparison to bath-type ultrasound, the application of horn-type ultrasound leads to significant changes in graphite particle size. Fig. 5a illustrates the effect of ultrasonic time on graphite particle size under the maximum ultrasound intensity (100%). Generally, as the sonication time increases, the peak of the PSD shifts towards smaller sizes. More specifically, after short periods of ultrasonic pretreatment (1 and 2 min), the peak of the PSD reduces from 150 μm to approximately 100 μm. However, when the ultrasonic pretreatment time is extended to 4 and 7 min, the peak of the PSD drops sharply to 80 μm and 60 μm, respectively. When the sonication time is fixed at 1 min, there is no significant variation in the PSD with an increase in sonication intensity from 20% to 80%. When the intensity reaches 100%, the PSD is slightly shifted towards smaller sizes, and the peak decreases to approximately 100 μm. Results suggest that higher ultrasonic intensity causes a significant reduction in graphite particle size. However, excessively fine graphite particles may not be conducive to the flotation performance. This may be a contributing factor to the inferior graphite flotation performance observed after prolonged treatment with high-intensity horn type ultrasound.

**Fig. 6:**
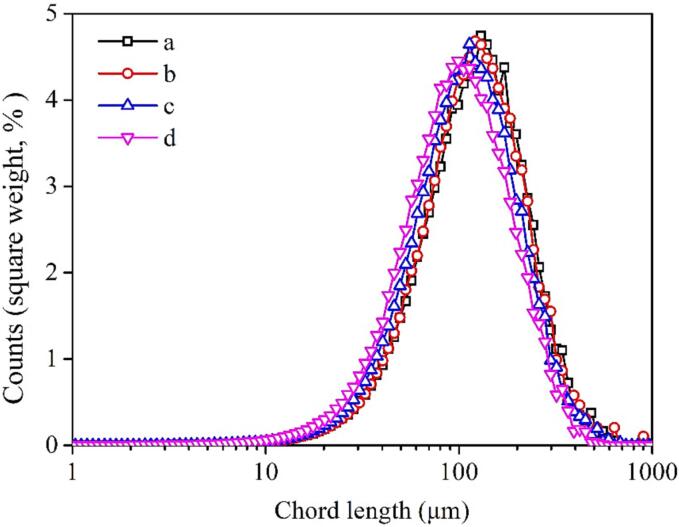
PSD of graphite after pretreated by different methods (a: without any treatment; b: bath-type ultrasound, intensity of 100 %, 4 min; c: horn-type ultrasound, intensity of 100%, 1 min; d: combination of ultrasounds with horn-type intensity of 100% and bath-type intensity of 60%, 1 min).

Fig. 6 depicts the PSD of graphite before and after undergoing the treatment of single-frequency and combination of two ultrasound types. The peaks of the PSD curve of graphite, in descending order, for the cases without any treatment/bath type ultrasound pretreatment, horn-type ultrasound pretreatment, and combination of ultrasounds pretreatment are 150, 100, and 90 μm, respectively. Results suggest that the reduction in graphite particle size achieved by the combination of ultrasounds is greater compared to that by single-frequency ultrasound.

#### 3.3.2 XRD and SEM-EDS analysis

**Fig. 7:**
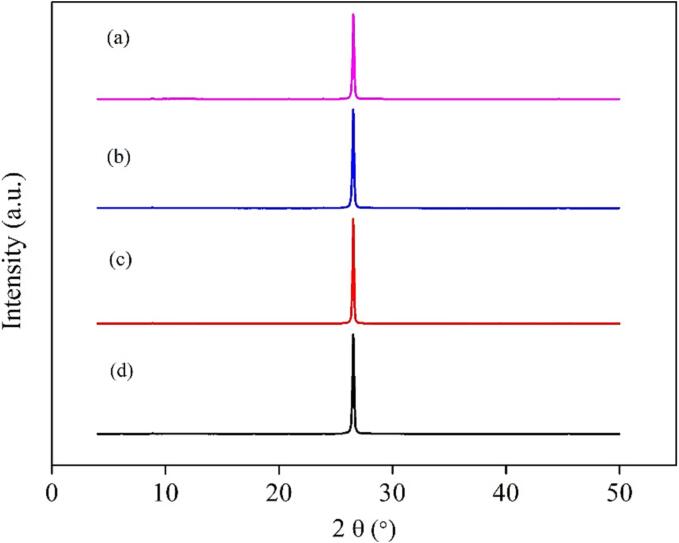
XRD data of the graphite flotation concentrate pretreated by different methods (a: graphite ore; b: horn-type ultrasound, intensity of 100%, 1 min; c: bath-type ultrasound, intensity of 100 %, 4 min; d: combination of ultrasounds with a horn-type ultrasound intensity of 100% and bath-type ultrasound intensity of 60%, 1 min).

To monitor changes in the properties and morphology of graphite samples resulting from ultrasonic pretreatment, and to further investigate the mechanisms underlying the effects of different ultrasonic pretreatments on graphite flotation recovery, XRD and SEM-EDS analysis were performed on the graphite flotation concentrates. The XRD results of the graphite concentrates obtained under different conditions are presented in Fig. 7. It is evident that the XRD patterns of the graphite concentrates all exhibit a single-peak distribution centered at approximately 26.5°, irrespective of the conditions. This finding suggest that the crystal structure of the graphite remained unchanged following ultrasonic pretreatment.

**Fig. 8:**
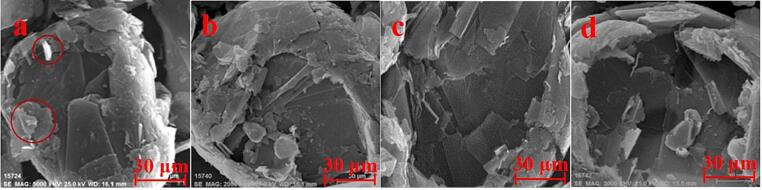
SEM images of graphite flotation concentrate pretreated by different methods (a: graphite ore; b: horn-type ultrasound, intensity of 100%, 1 min; c: bath-type ultrasound, intensity of 100 %, 4 min; d: combination of ultrasounds with horn-type ultrasound intensity of 100% and bath-type ultrasound intensity of 60%, 1 min).

Fig. 8 depicts the SEM images of graphite flotation concentrate subjected to different treatments. Unlike XRD results, the surface morphology of the flotation concentrates obtained from different treatments exhibited significant differences. The SEM micrograph revealed that the raw graphite ore is a typical flake graphite with a relatively rough surface and dull luster (Fig. 8a), which is likely due to the coating of some fine gangue minerals. However, ultrasonic pretreatment induced substantial changes in the morphology of the graphite flotation concentrate, and changes varied depending on the processing method employed. In general, all ultrasonic pretreatments altered the graphite surface morphology to some degree, with the bath-type ultrasonic having the least effect, followed by the horn-type, and the combination of ultrasonic pretreatment exerting the largest impact. Specifically, the horn-type ultrasound pretreatment resulted in the appearance of pits with a size of about 30 × 30 μm on the graphite surface, exposing fresh surfaces (Fig. 8b). Bath types of the ultrasound pretreatment only partially cleaned the graphite surface, and no large pits were observed (Fig. 8c). In contrast, the pretreatment with the combination of ultrasounds led to the formation of larger pits (with a size of 90 × 90 μm) on the graphite surface (Fig. 8d), the exfoliation of the graphite surface by ultrasound can be attributed to the collapse of ultrasonic cavitation bubbles. In recent years, some in-situ characterization techniques, such as Ultrafast Synchrotron X-ray imaging and high-speed optical images, have been applied to monitor the interaction between ultrasonic cavitation bubbles and graphite [41], [42]. Their findings suggest that the shock wave resulting from bubble implosion can produce sufficient stresses to cause microsecond fatigue exfoliation of graphite layers [43], [44]. Moreover, controlling the number density of bubbles in the cavitation zone can accelerate the exfoliation rate tens or even hundreds of times, achieved by optimizing ultrasound parameters to maximize cavitation bubbles [44]. Additionally, Morton, et al. [45] discovered that the dual-frequency system can enlarge the cavitation cloud size and intensity, potentially explaining why the graphite surface exfoliation caused by dual-frequency ultrasound in our experiment is more pronounced.

**Fig. 9:**
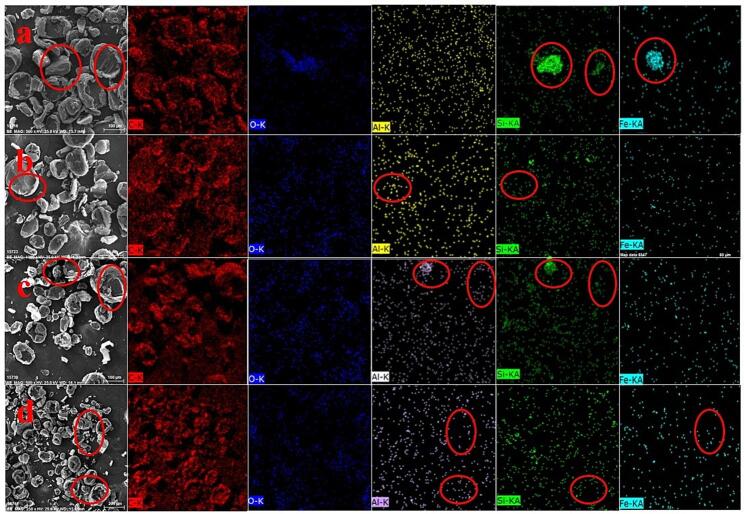
SEM-EDS images of graphite flotation concentrate pre-treated by different methods. (a: graphite ore; b: horn-type ultrasound, intensity of 100%, 1 min; c: bath-type ultrasound, intensity of 100 %, 4 min; d: Combination of two types of ultrasounds with the horn-type ultrasound intensity of 100% and bath-type ultrasound intensity of 60%, 1 min).

Furthermore, the distribution of elements analyzed on the surface of the graphite flotation concentrate obtained under different conditions was examined. The elements analyzed in this experiment were C, Si, O, Al, and Fe, which are the main elements of gangue minerals such as quartz and kaolinite. As shown in Fig. 9a, the Si, O, Al, and Fe elements in the raw graphite ore are distributed in flakes, indicating that gangue minerals are embedded in the graphite ore, and their distribution is relatively concentrated. The pretreatment by the bath-type ultrasound did not completely eliminate the embedded gangue minerals, as evidenced by the planar distribution of Al and Si elements (Fig. 9c). Conversely, the treatment by the horn-type or combination of two types of ultrasounds liberated the embedded gangue minerals to some extent, resulting in a spotty distribution of the analyzed elements in the flotation concentrate (Fig. 9b and d).

### 3.4 Discussions

**Fig. 10:**
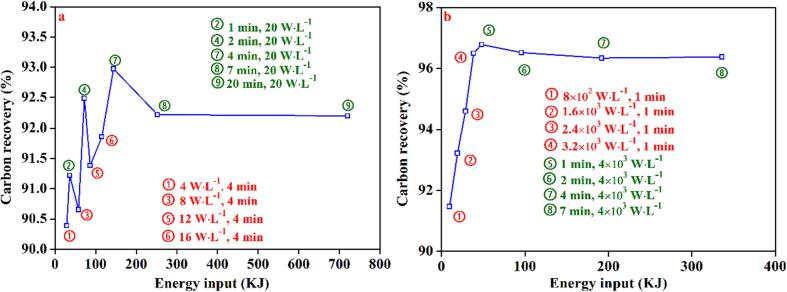
Carbon recovery as a function of energy consumption (a: bath-type; b: horn-type).

In this study, the energy input was modulated by manipulating the ultrasonic radiation duration and intensity. Fig. 10 depicts the carbon recovery of graphite flotation as a function of energy input. Experimental findings demonstrate an increasing and then decreasing trend in the carbon recovery rate with escalating energy input for both bath- and horn-type ultrasounds. This implies that excessive energy input hinders the effective flotation, irrespective of the ultrasound type. However, the curve of bath-type ultrasound fluctuates more. Data analysis showed that, despite increased energy input, merely prolonging low-intensity ultrasound exposure time did not lead to good results. By contrast, the curve generated under the experimental condition with the horn-type ultrasound displays a promising trend, probably because tests done with the horn-type ultrasound were not configured at a low intensity for an extended duration, but it is expected to exhibit the same trend as with the bath-type ultrasound. This is because at a lower intensity, irrespective of the ultrasound type, the cavitation intensity is low, and extending the duration does not engender appropriate cavitation intensity, leading to weaker particle cleaning or crushing.

The impact of ultrasound type on the carbon recovery during graphite flotation exhibits significant variability. While differences exist in the frequency and power of the two ultrasound types, they were not considered to be the primary cause of the difference in carbon recovery. Thus, differences in graphite flotation caused by bath-type and horn-type ultrasound can be attributed to two factors. Firstly, the bath-type ultrasound generates a relatively uniform sound field, which uniformly transmits sound waves to every part of the slurry, resulting in a relatively low energy radiation received per unit volume of the slurry. As a result, graphite particles are not broken by the ultrasound, and their surface may only be simply cleaned in a short radiation time. Over-dispersion of the pulp may also occur if the ultrasound treatment time is too long, which may contribute to the poor flotation performance after longer exposure to bath-type ultrasound. On the other hand, the probe for the horn-type ultrasound can act directly on the slurry, it will result in a significantly higher ultrasonic radiation energy per unit volume of pulp compared to the bath-type ultrasound, causing instantaneous release of energy that produces high-speed shock waves and micro jets locally. These strong mechanical effects will, to some extent, liberate the gangue minerals embedded in the graphite particles, thereby improving the selectivity of graphite flotation. However, over-crushing of graphite particles may occur when the ultrasonic treatment time is too long, which can be detrimental to their flotation recovery.

**Fig. 11:**
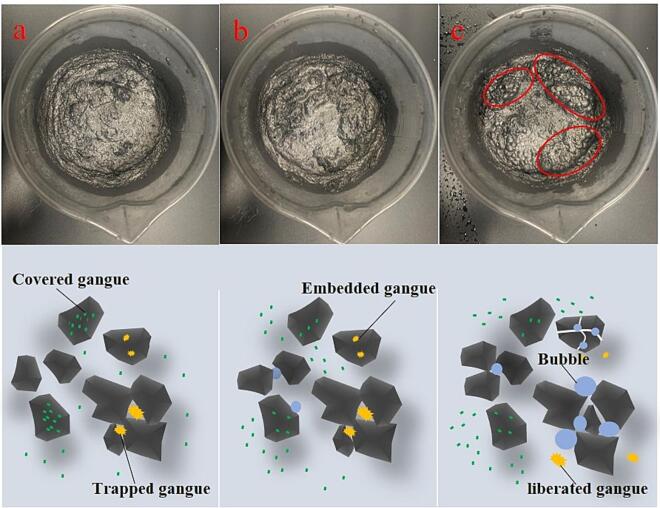
Graphite slurry after pretreated by different methods (a: without pretreatment; b: bath-type ultrasound with intensity of 100%, 4 min; c: horn-type ultrasound with intensity of 100%, 1 min).

Secondly, the structure of slurry changes differently when the slurry is pretreated by these two types of ultrasounds. For untreated graphite samples, some unqualified graphite concentrates, such as graphite covered by gangue minerals and graphite particles with a small number of embedded gangue minerals, are present (Fig. 11a). In addition, due to the strong hydrophobicity of graphite itself, aggregates are formed when dispersed in water, and some gangue particles are wrapped in the aggregates, which is unfavorable for further flotation separation. When the slurry is pretreated by bath-type ultrasound (Fig. 11b), some graphite particles covered with gangue minerals are cleaned, and some gases dissolved in the slurry are excited to nucleate on the surface of hydrophobic graphite. However, this type of ultrasound cannot liberate the gangue minerals embedded in the graphite. On the other hand, the horn-type ultrasound not only cleans the mineral surface but also liberates gangue particles embedded in graphite due to its strong cavitation effect. Additionally, the strong cavitation effect breaks up the flocs to release the gangue particles and reorganizes the graphite flocs. Moreover, the strong cavitation effect stimulates the release of dissolved gas in the water to form cavitation bubbles that carry graphite particles to form gas flocs floating on the surface of the slurry (Fig. 11c), which is highly beneficial for further enrichment of graphite concentrate.

Based on results obtained from the aforementioned experiments, it is evident that various operational parameters employed during ultrasonic pretreatment can significantly impact properties of graphite particles. Such alterations in particle properties may prove to be a critical factor affecting the flotation performance of graphite. Nonetheless, it appears that the energy input magnitude is not the principal determinant governing graphite flotation. In order to provide further insight into the influence of different operational parameters or application modes on graphite flotation during sonication, the cavitation intensity was monitored under certain sonication conditions. Results of the tests conducted are presented in Table 6. In general, the concentration of I_3_^−^ gradually increased as the ultrasound time increased for both single-frequency and combined-frequency ultrasound, which also suggests that an increase in ultrasound time promotes a corresponding increase in cavitation intensity at the maximum ultrasound intensity. However, the increase in cavitation intensity with time remained slow for all three ultrasound modes of action when the ultrasound time was less than four minutes. Notably, the cavitation intensity of horn-type ultrasound and combination of two types of ultrasounds showed a sharp rise once the ultrasound duration reached seven minutes. Under the same ultrasonic treatment time, the I_3_^−^ concentrations ranked from low to high were the bath-type ultrasound, horn-type ultrasound, and combination of two types of ultrasounds, with the I_3_^−^ concentration with the combination of ultrasounds exceeding that of the other two single-frequency ultrasound. This implies that combination of ultrasounds exhibits the strongest cavitation intensity, which also accounts for the distinct changes observed in particle size and morphology of graphite (Fig. 6 – Fig. 9) after three different ultrasound treatments.

**Table 6:** Effect of ultrasonic operating parameters on the cavitation intensity.

Time /min	Bath type intensity / %	I_3_^−^ concentration / mM	Horn type intensity / %	I_3_^−^ concentration / mM	Combined Frequency (Bath/Horn)	I_3_^−^ concentration / mM
1	100	6.10 × 10^−3^	100	3.84 × 10^−2^	20/100	7.43 × 10^−2^
2	100	7.54 × 10^−3^	100	4.85 × 10^−2^	20/100	8.29 × 10^−2^
4	100	9.69 × 10^−3^	100	6.35 × 10^−2^	20/100	9.51 × 10^−2^
7	100	1.18 × 10^−2^	100	0.12 × 10^−2^	20/100	2.24 × 10^−2^

The influence of ultrasound on graphite particles has been investigated, and it has been found that the combination of ultrasounds helps produce the highest cavitation intensity compared to single frequency ultrasound when sonicated for a short duration of 1 min. This leads to effective liberation of the gangue in graphite particles without causing significant damage to the graphite particle size, thus facilitating further enrichment of graphite. However, prolonged sonication with the combination of ultrasounds results in severe fragmentation of the graphite flakes due to the intense cavitation, which outweighs the benefits of enhanced gangue liberation. Consequently, it is imperative to carefully regulate the cavitation intensity to ensure optimal conditions for ultrasound application in graphite flotation.

## 4 Conclusions

(1) In the context of graphite flotation, the type of ultrasound employed exerts a substantial influence on the carbon recovery of the process when utilizing single frequency ultrasound. Specifically, the use of bath-type ultrasound yields only a modest improvement in the graphite carbon recovery, with a maximum enhancement of approximately 2%. In contrast, the implementation of horn-type ultrasound results in a significant increase in the graphite carbon recovery, with a maximum enhancement of around 7%. The enhanced performance with the bath-type ultrasound is primarily attributed to the removal of gangue minerals that are obstructing the graphite surface. In comparison, the horn-type ultrasound facilitates not only the cleaning of the graphite surface but also the liberation of gangue embedded in the graphite particles. Consequently, the graphite particle size reduces after the horn-type ultrasound pretreatment, while no substantial change in the particle size is observed after the treatment of bath-type ultrasound.
(2) The efficiency of both types of single-frequency ultrasound in enhancing graphite flotation performance is influenced by two main factors, namely the duration of ultrasound exposure and the intensity of ultrasound. Specifically, the improvement in graphite flotation performance exhibits an increasing-then-decreasing trend as the duration of ultrasonic radiation increases. Moreover, the graphite flotation performance gradually improves with increasing the ultrasonic intensity. Notably, the recovery of graphite carbon is not strictly proportional to the total ultrasonic input energy. For instance, at lower ultrasonic intensities, extending the duration of ultrasonic radiation alone does not sufficient to enhance graphite flotation. These findings suggest that instantaneous high-intensity cavitation is beneficial to graphite flotation, but the duration of this high-intensity ultrasonic radiation must be carefully regulated, as prolonged ultrasonic exposure can severely compromise graphite particle size and thereby undermine the graphite flotation performance.
(3) In contrast to single-frequency ultrasound, the combination of ultrasounds exhibits a greater cavitation intensity. However, it does not demonstrate a cooperative effect for the carbon recovery, and the preponderant influence of the combination system of ultrasounds stems from the intensity of horn-type ultrasound. Under particular conditions, where the duration of ultrasound radiation is held constant, the carbon recovery of graphite after the treatment of the combination of ultrasounds surpasses that of single-frequency ultrasound, albeit with negligible improvement. However, the extensive and intense cavitation of the combination of ultrasounds leads to significant reductions in graphite particle size during extended radiation periods, thereby negatively affecting graphite flotation.

## CRediT authorship contribution statement

**Shaoqi Zhou:** Methodology, Software, Validation, Investigation, Writing – original draft, Writing – review & editing. **Zheng Tong:** Methodology, Investigation, Formal analysis. **Lisha Dong:** Formal analysis, Writing – original draft, Writing – review & editing. **Xiangning Bu:** Conceptualization, Visualization, Project administration, Funding acquisition, Writing – review & editing. **Chao Ni:** Methodology, Data curation. **Guangyuan Xie:** Resources, Supervision. **Muidh Alheshibri:** Project administration, Writing – original draft, Writing – review & editing.

## Declaration of Competing Interest

The authors declare that they have no known competing financial interests or personal relationships that could have appeared to influence the work reported in this paper.

## Data Availability

Data will be made available on request.
